# Development and validation of a combined cuproptosis and immunogenic cell death prognostic model for diffuse large B-cell lymphoma

**DOI:** 10.18632/aging.205399

**Published:** 2024-01-26

**Authors:** Nana Wang, Shanshan Shi, Moran Li, Xiaoning Yu, Guangxin Ma

**Affiliations:** 1 Department of Hematology, Qilu Hospital of Shandong University, Jinan 250012, China; 2 Department of Geriatrics, Hematology and Oncology Unit, Qilu Hospital of Shandong University, Jinan 250012, China

**Keywords:** cuproptosis, immunogenic cell death, lncRNA, prognostic model, DLBCL

## Abstract

Background: Diffuse large B-cell lymphoma (DLBCL) is the most common type of non-Hodgkin lymphoma worldwide with a high degree of heterogeneity. Cuproptosis and immunogenic cell death (ICD) have been considered to be vital for tumor progression. However, current understanding of cuproptosis and immunogenic cell death in DLBCL is still very limited. We aim to explore a prognostic model combining cuproptosis and immunogenic cell death in DLBCL.

Methods: Pearson’s correlation analysis was utilized to acquire lncRNAs associated with cuproptosis and immunogenic cell death. Prognostic biomarker identification and model construction involved the use of univariate Cox regression, least absolute shrinkage and selection operator (LASSO) Cox regression, and multivariate Cox regression. We assessed the predictive capability of the risk model by conducting Kaplan-Meier analysis and time-dependent ROC analysis. The analysis and comparison of immune infiltration and drug sensitivity were conducted in this study. Moreover, RT-qPCR was employed to validate the expression of lncRNAs associated with cuproptosis and immunogenic cell death in DLBCL cell lines.

Results: We identified 4 prognosis-related lncRNAs (ANKRD10-IT1, HOXB-AS1, LINC00520 and LINC01165) that were correlated with cuproptosis and immunogenic cell death. The model was verified to have a good and independent predictive ability in the prognostic prediction of DLBCL patients. Moreover, significant difference was observed in immune infiltration and drug sensitivity between high- and low-risk groups.

Conclusion: Our discoveries could enhance the comprehension of the role of cuproptosis and ICD in DLBCL, potentially offering novel viewpoints and knowledge for personalized and precise treatment of DLBCL individuals.

## INTRODUCTION

DLBCL is the most common type of non-Hodgkin lymphoma worldwide with a high degree of heterogeneity, representing almost 30% of all cases [1–3]. Despite most DLBCL patients achieve complete remission (CR) with standard treatment rituximab plus cyclophosphamide, doxorubicin, vincristine, and prednisone (R-CHOP), still 40% of patients ultimately suffer from relapsed or refractory disease [4, 5]. Thus, discovery of new drugs and the proposal of novel treatment are urgently needed for relapsed or refractory DLBCL patients.

As a crucial trace element, copper is maintained at relatively low levels in mammalian cells and regulates various physiological processes. It also exhibits cytotoxicity when intracellular concentrations of copper ions exceed the threshold for maintaining homeostatic mechanisms [6–8]. Disturbances of copper homeostasis have been associated with many diseases, including malignant tumors [9]. Moreover, increasing evidence connects copper signaling to tumor growth and metastasis, representing the therapeutic potential of targeting copper homeostasis [6]. Cuproptosis is a controlled type of cell death that is dependent on copper and requires mitochondrial respiration. Accumulation of copper inside cells triggers the aggregation of lipoylated enzymes in the tricarboxylic acid (TCA) cycle through copper binding. This leads to cell death caused by proteotoxic stress, known as cuproptosis [10].

ICD has been identified as a type of regulated cell death with different functions, which can stimulate an immune response against dead-cell antigens, in particular cancer cells [11]. A series of signaling molecules were generated with the emergence of ICD in tumor cells, which are called damage associated molecular patterns (DAMPs) [12]. The DAMPs include secreted ATP, surface-exposed calreticulin (CRT) and released high mobility group protein B1(HMGB1), which was reported as a key immune regulator of cuproptosis-initiated sterile inflammation in recent reports [13]. By binding to the pattern recognition receptors (PRRs) on the surface of DC cells, DAMPs have the ability to trigger a cascade of cytological reactions, leading to the activation of both innate and adaptive immune responses [14]. Additionally, the administration of chemotherapeutic medications leads to ICD, thereby boosting the immune response against tumors [15]. Previous researchers reported that therapeutic regimens based on the copper or copper chelation could effectively induce ICD and have the potential function in antitumor immunity [16, 17]. The accumulated evidence suggests that an underlying connection may exist between the cuproptosis and ICD. Nevertheless, little focus has been given to investigating this correlation.

The study involved the examination of prognostic lncRNAs associated with cuproptosis and immunogenic cell death. Additionally, a risk signature was developed using the identified prognostic lncRNAs. We also evaluated its prognostic prediction performance of DLBCL patients. Furthermore, we extensively examined the association between the risk model and immune infiltration, clinicopathologic features, and drug response treatment.

## MATERIALS AND METHODS

### Data collection

The gene expression profile of DLBCL patient samples were obtained from GEO (https://www.ncbi.nlm.nih.gov/geo/) databases. Utilizing the Perl script, we extracted the gene expression file of each DLBCL samples and merged into a file. Corresponding DLBCL clinical information was also acquired from the GEO database using Perl scripts and subsequently merged into the final file. A total of 846 DLBCL samples were collected from three cohorts, including GSE11318 (*n* = 203), GSE10846 (*n* = 420) and GSE87371 (*n* = 223). After excluding samples with incomplete transcriptomic and clinical information, we obtained 414 DLBCL samples for next study.

### Identification of cuproptosis-related lncRNAs and immunogenic cell death-related lncRNAs

From the previous study [10], a total of 10 genes associated with cuproptosis (CRGs) were gathered (Supplementary Table 1). Besides, a recent investigation [18] provided 33 genes related to immunogenic cell death (IRGs) (Supplementary Table 2). Pearson correlation algorithm was utilized to calculate the coefficient of genes and lncRNAs. A total of 170 cuproptosis-related lncRNAs (CRLs) and 258 immunogenic cell death-related lncRNAs (IRLs) were identified for subsequent analysis based on the selection threshold of |correlation coefficient| > 0.4, *p*-value < 0.001. Venn analysis was used to obtain the overlapping genes from two analyses above.

### Development and verification of a risk model for DLBCL patients based on lncRNAs associated with cuproptosis and immunogenic cell death (CRIRLs)

To assess the predictive significance of CRIRLs, the prognostic value was evaluated using both the univariate Cox regression and LASSO algorithm. Furthermore, the multivariate Cox analysis was employed to determine the independent prognostic characteristic of CRIRLs. A risk model was created using these chosen CRIRLs to forecast the outcome of DLBCL. The risk score for every individual was computed by utilizing the subsequent equation: risk score = (−2.69) × ANKRD10-IT1 expression + (−1.15) × HOXB-AS1 expression + (2.99) × LINC00520 expression + (0.66) × LINC01165 expression. The risk score’s median value was used as the cutoff to cluster groups into high-and low-risk categories. Kaplan-Meier survival analyses were used to estimate the disparities in overall survival (OS) between the high- and low-risk groups. Furthermore, we performed principal component analysis (PCA) and t-SNE analysis to assess the clustering capability of the risk signature utilizing the “Rtsne” and “ggplot2” package. The group of samples used for training consisted of 290 DLBCL samples, whereas the validation group consisted of 124 DLBCL samples obtained from the GEO database. The risk score for each sample was calculated in both cohorts, and then the samples were divided into low-and high-risk groups using the median risk score. Supplementary Tables 3–5 contained the complete dataset, internal training set, and validation set, respectively.

### Analysis of the potential clinical relevance of the prognostic signature

We performed univariate and multivariate Cox regression analysis to select independent indicators of DLBCL from several clinicopathological features and CRIRLs risk model. Besides, we employed the “pROC” package to examine the diagnostic power of risk score and clinicopathological characteristics.

### Immune infiltration level analysis

We explored the immune status of DLBCL samples based on the ESTIMATE algorithm using “estimate” R package. Moreover, ssGSEA algorithm was utilized to evaluate the immune microenvironment characteristic of DLBCL samples using the “GSVA” R packages.

### Drug sensitivity analysis

Using the R package “pRRophetic”, the Genomics of Drug Sensitivity in Cancer (GDSC) database was utilized to determine the drug sensitivity (IC50) and predict the drug response of each sample in both low- and high-risk groups. The “ggplot2” R package was used to visualize all statistical analyses.

### Cell culture

The GM-12878 cell line, a human B lymphoid cell line [19], along with the DB, RC, and U2932 cell lines, which are human DLBCL cell lines, were acquired from the American Type Culture Collection (ATCC) located in Manassas, VA, USA. The cells were grown in RPMI-1640 medium from Gibco/BRL (New York, NY, USA), supplemented with 10% FBS from Gibco (New York, NY, USA), and incubated at 37°C in a 5% CO2 humidified atmosphere.

### RNA extraction and qRT-PCR

In this study, total RNA was isolated by TRIZOL (Thermo Fisher Scientific, Waltham, MA, USA) and Bestar^™^ qPCR RT Kit (DBI Bioscience, Newark, DE, USA) was used for cDNA synthesis following the manufacturer’s instructions from cell lines. To standardize the reactions, the GAPDH gene was employed as an internal control. Four lncRNA gene relative expressions were calculated by the 2^−ΔΔCt^ method. The primer sequences of four lncRNA were shown in Supplementary Table 6.

### Statistical analysis

The statistical analysis in this study was conducted using R software and Perl scripts. To assess the accuracy of the prognostic CRIRLs, we employed Spearman-ranked correlation analysis, considering statistical significance as *P* < 0.05.A Wilcoxon rank-sum test was performed to examine the contrasting functions between the high-risk and low-risk groups. Statistical significance was determined by a *P*-value less than 0.05.

### Data availability

All data generated or analyzed during this study are included in this published article.

## RESULTS

### Construction of prognostic risk model for patients with DLBCL based on the CRIRLs

**Figure 1:**
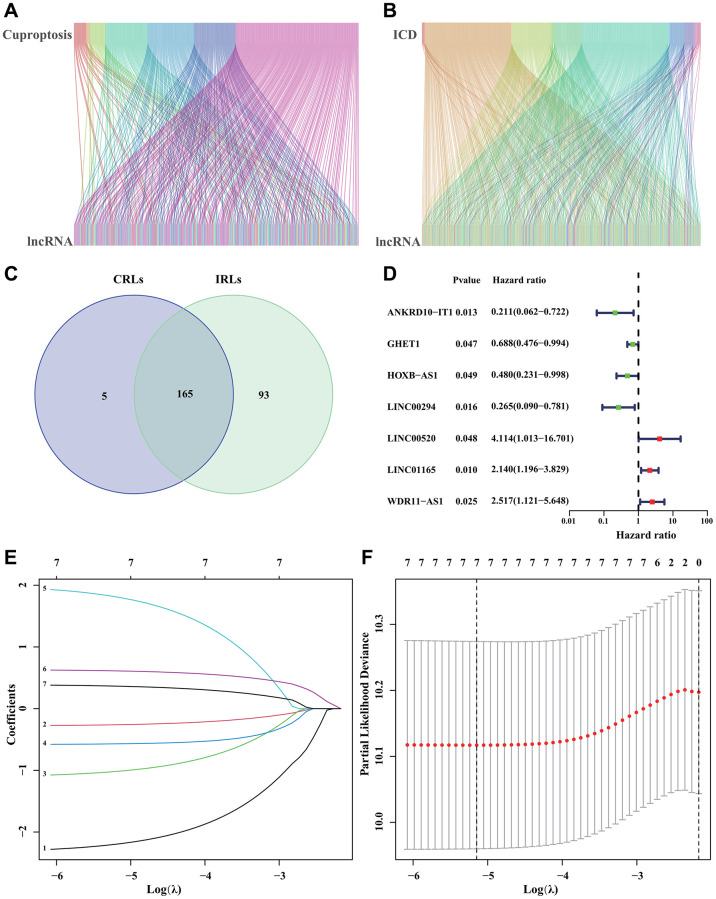
**Identification of CRIRLs.** (**A**) Sankey chart illustrating the relationship between CRGs and CRLs. (**B**) Sankey diagram of relationship between IRGs and IRLs. (**C**) Venn diagram of CRLs and IRLs. (**D**) The prognostic CRIRLs were analyzed using univariate Cox regression. (**E**, **F**) LASSO regression analysis displays the minimum lambda and optimal coefficients of prognostic CRIRLs.

**Figure 2:**
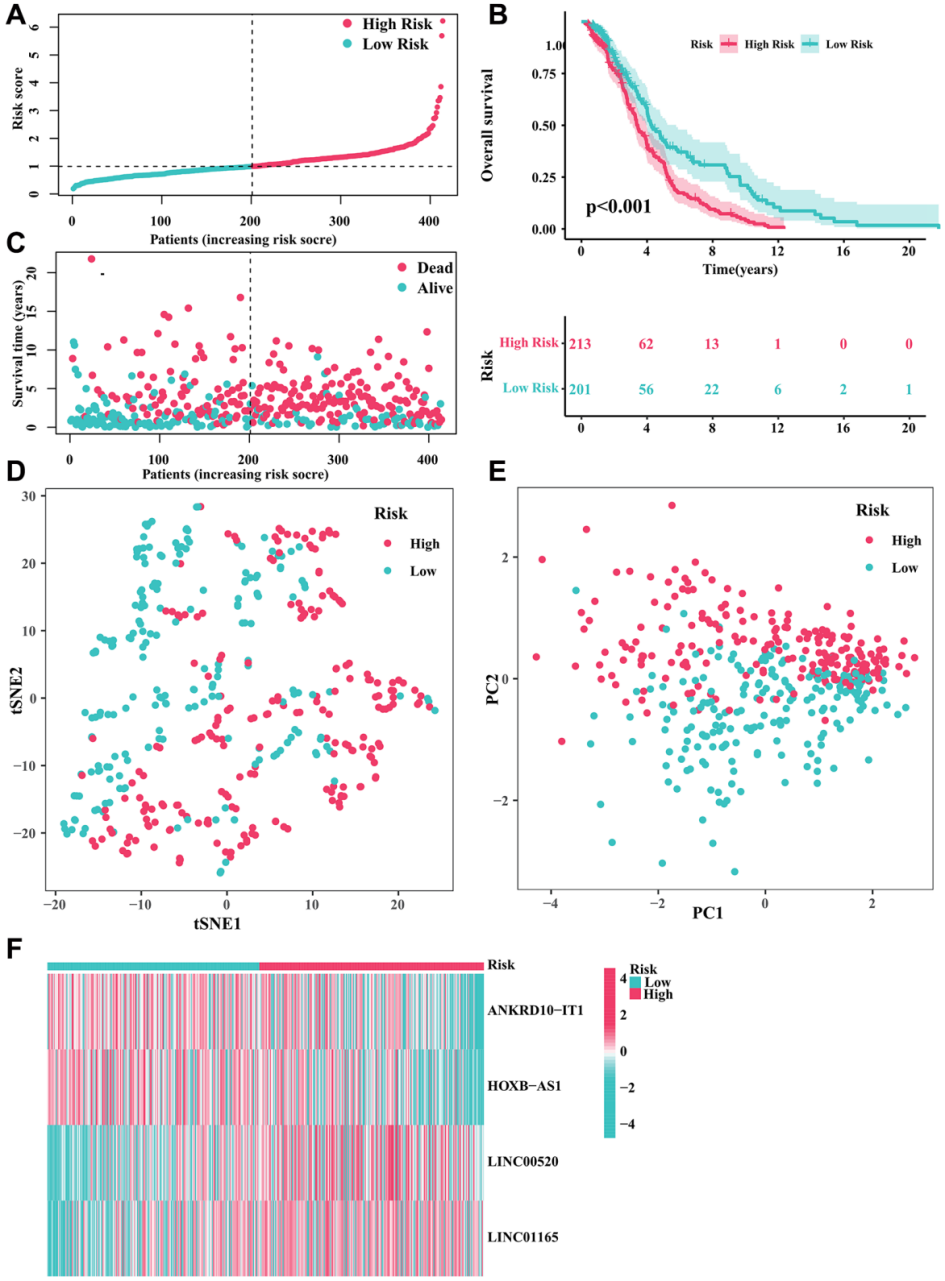
**Developing a predictive risk model using the CRIRLs in DLBCL.** (**A**) Distribution of the patients’ normalized risk score. (**B**) Analysis of clinical prognosis in low-risk and high-risk groups of DLBCL samples. (**C**) Patients’ survival status along with their risk score. (**D**) t-SNE analysis. (**E**) PCA analysis. (**F**) The heatmap diagram of the expression of 4 prognostic CRIRLs.

Figure 1A depicted the correlation between CRGs and CRLs. In the meantime, the correlation between IRGs and IRLs was illustrated in Figure 1B. Figure 1C displayed a Venn diagram showing that a grand total of 165 CRIRLs were selected for additional investigation. Using the LASSO analysis, 7 prognostic CRIRLs were identified through univariate Cox regression analyses (Figure 1D–1F). Next, the multivariate Cox regression analysis was used to select 4 CRIRLs that provided the best prognostic value for establishing the risk model. The DLBCL samples were categorized into two groups based on their risk levels, namely low-risk and high-risk. Figure 2A, 2C displayed a scatter dot plot indicating an inverse correlation between the risk score and the overall survival (OS) of DLBCL patients. According to Figure 2B, the Kaplan–Meier curve demonstrated that patients with high-risk scores had a considerably shorter overall survival (OS) compared to those with low-risk scores. By employing t-SNE (Figure 2D) and PCA (Figure 2E) analysis, we examined the distribution of two distinct risk categories. The findings revealed a notable segregation of DLBCL patients into low- and high-risk groups. The heatmap diagram clearly showed the expression of 4 prognostic CRIRLs in the two different risk groups (Figure 2F).

### Validation of the prognostic risk signature

**Figure 3:**
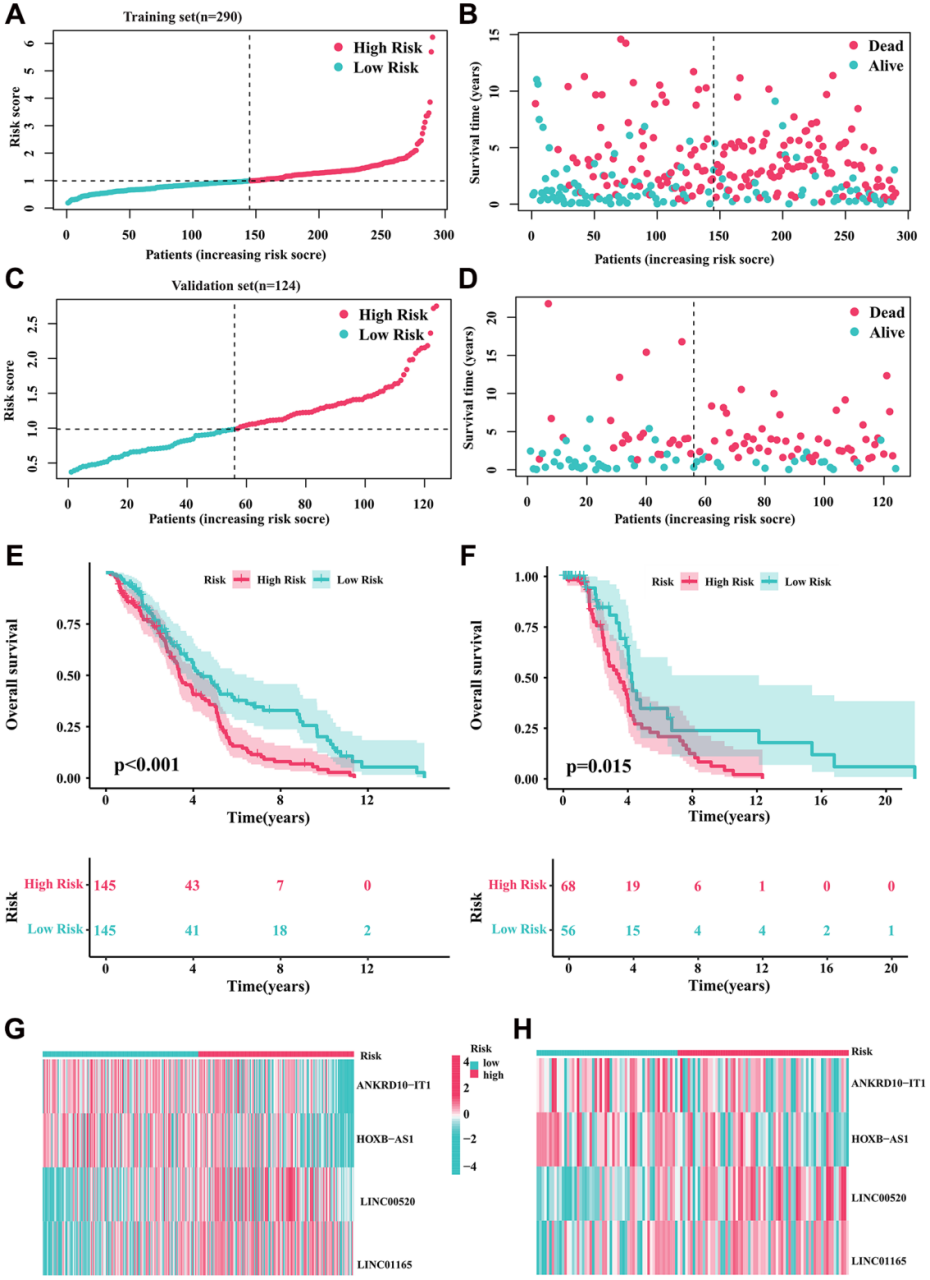
**Validation of the prognostic risk signature.** (**A**, **B**) Stratification of CRIRLs into risk subgroups in training cohort. (**C**, **D**) Stratification of CRIRLs into risk subgroups in validation cohort. (**E**, **F**) Analysis of clinical prognosis outcomes for risk subgroups in training cohort and validation set of CRIRLs. (**G**, **H**) Expression of the 4 prognostic CRIRLs in training cohort and validation set.

For internal validation, we randomly divided all DLBCL samples into training and validation cohorts to evaluate the CRIRLs prognostic signature’s ability to predict the prognosis of DLBCL patients. The results of our study showed that the clinical prognostic results of the DLBCL samples in the low-risk category were considerably superior to those in the high-risk category in the training set (Figure 3A, 3B, 3E). Moreover, within the internal validation set, we consistently observed that DLBCL samples belonging to the high-risk subgroup displayed a more unfavorable clinical prognosis compared to those in the low-risk subgroup (as shown in Figure 3C, 3D, 3F). The high-risk group exhibited notably elevated levels of LINC00520 and LINC01165 expression, whereas ANKRD10-IT1 and HOXB-AS1 expression was decreased in both cohorts of the high-risk group (Figure 3G, 3H). Table 1 provides an outline of the baseline characteristics of patients in both the complete and internal training and validation cohorts.

**Table 1:** The clinical characteristics of patients in different cohorts.

**Variable**	**Total (*n* = 414)**	**Train (*n* = 290)**	**Validate (*n* = 124)**	**Statistic**	***P***
Gender, *n* (%)					
Female	172 (43.43)	123 (44.40)	49 (41.18)	χ² = 0.353	0.552
Male	224 (56.57)	154 (55.60)	70 (58.82)		
Age, M (Q_1_, Q_3_)	62.50 (52.00–73.75)	64.00 (52.00–74.00)	59.00 (v49.00–72.00)	Z = 2.058	0.040
LDH ratio, M (Q_1_, Q_3_)	1.01 (0.77–1.62)	1.01 (0.78–1.68)	1.01 (0.75–1.44)	Z = 0.419	0.675
Clinical diagnosis, *n* (%)					
ABC DLBCL	167 (40.34)	123 (42.41)	44 (35.48)	χ² = 2.809	0.245
GCB DLBCL	183 (44.2)	127 (43.79)	56 (45.16)		
Unclassified DLBCL	64 (15.46)	40 (13.79)	24 (19.35)		
ECOG performance status, *n* (%)					
0	85 (21.85)	57 (21.03)	28 (23.73)	χ² = 4.870	0.301
1	211 (54.24)	152 (56.09)	59 (50.00)		
2	60 (15.42)	37 (13.65)	23 (19.49)		
3	28 (7.2)	20 (7.38)	8 (6.78)		
4	5 (1.29)	5 (1.85)	0 (0.00)		
Stage, *n* (%)					
1	66 (16.26)	45 (15.79)	21 (17.36)	χ² = 1.623	0.654
2	122 (30.05)	83 (29.12)	39 (32.23)		
3	97 (23.89)	73 (25.61)	24 (19.83)		
4	121 (29.8)	84 (29.47)	37 (30.58)		
Risk score, M (Q_1_, Q_3_)	1.00 (0.73–1.36)	0.99 (0.76–1.35)	1.06 (0.71–1.36)	Z = 0.339	0.734

### Survival analysis of CRIRLs prognostic signature in different clinicopathological characteristics

**Figure 4:**
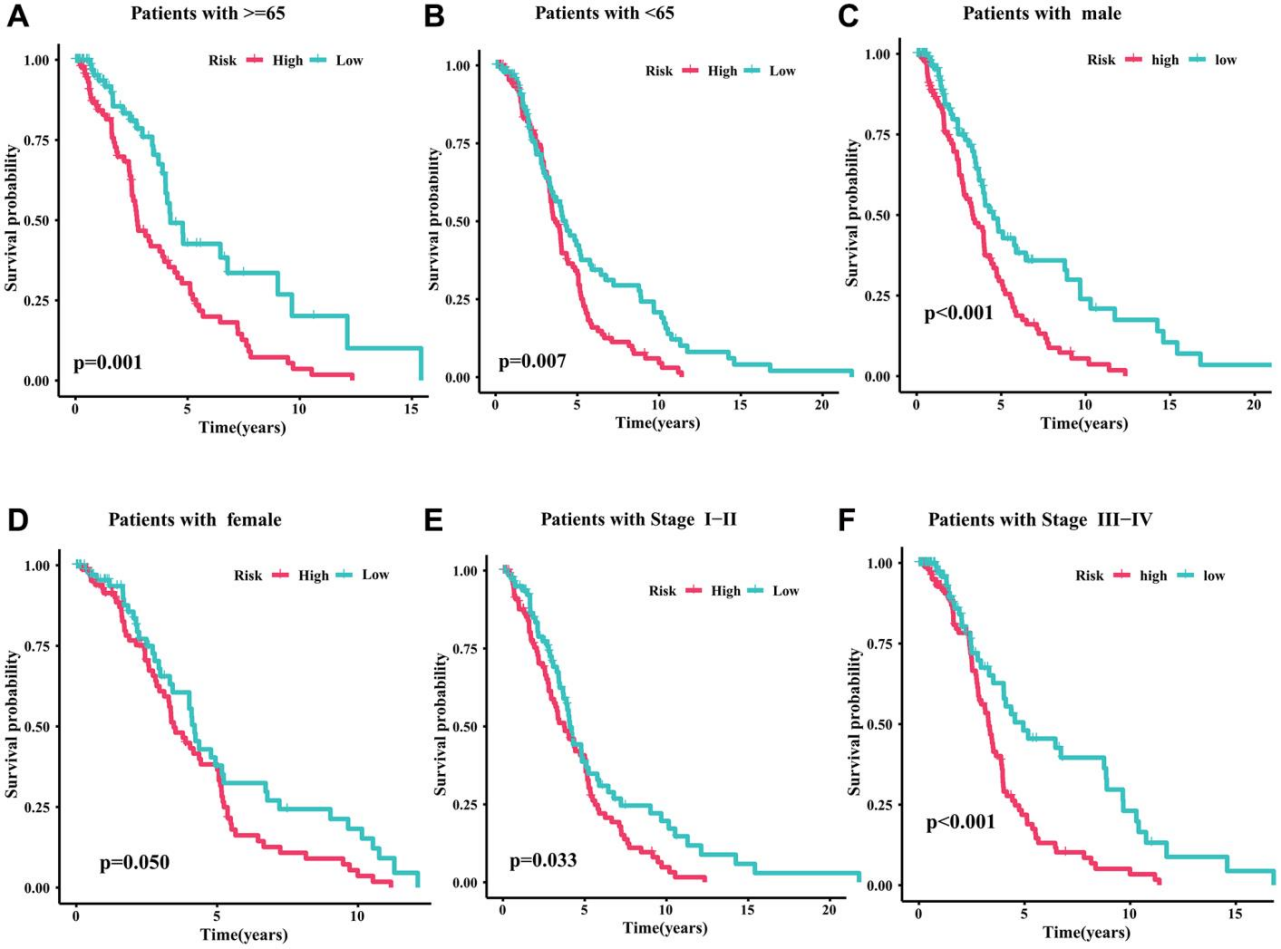
**Survival analysis of CRIRLs prognostic signature.** (**A**) Age ≥65 (**B**) Age <65 (**C**) Male (**D**) Female (**E**) Stage I+II (**F**) Stage III+IV.

To evaluate the clinical application value of CRIRLs prognostic signature, the prognostic value of the CRIRLs prognostic signature were explored by subgroup analysis. Based on the risk score, DLBCL patients were categorized into the low- and high-risk groups among the different clinicopathological features. As summarized in Figure 4, among DLBCL patients with age ≥65, age <65, male, female, stage I-II and stage III-IV, the OS of the high-risk group was significantly lower than that of the low-risk group (Figure 4A–4F). In short, these results demonstrate accuracy of CRIRLs prognostic signature in prognostic prediction for DLBCL patients.

### Independent prognostic analysis of CRIRLs score

**Figure 5:**
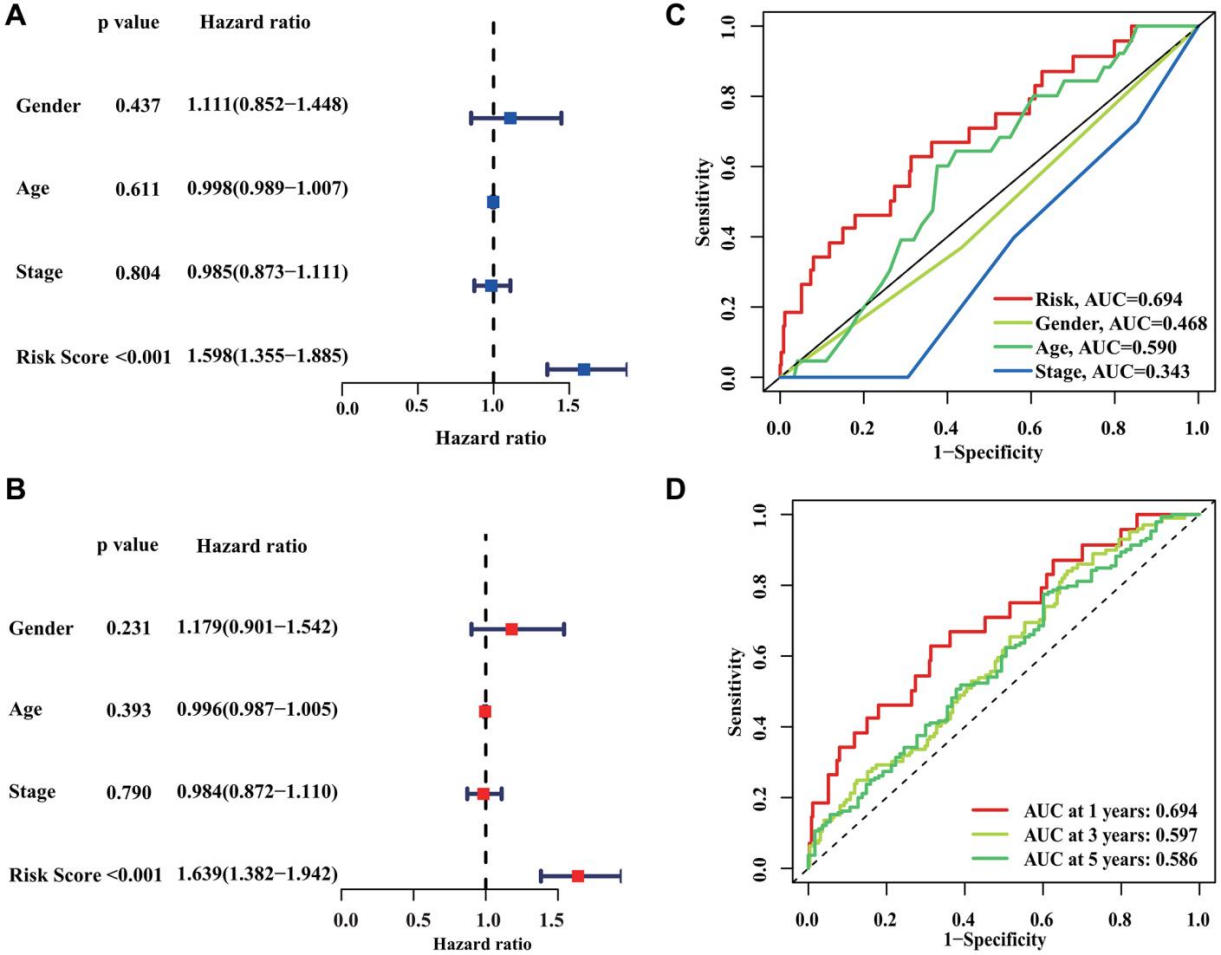
**Independent prognostic analysis of CRIRLs score.** (**A**, **B**) HR and *P* values of CRIRLs score and different clinical pathological features were evaluated based on univariate and multivariate Cox analyses. (**C**) Diagnostic effectiveness evaluation of CRIRLs score and clinical pathological features. (**D**) Time-dependent ROC curve shows the 1-, 3-, and 5-year AUC.

Further analysis was performed to evaluate the independent predictive significance of the risk model created using the prognostic CRIRLs along with other clinic-pathological features. The findings of the Univariate COX analysis indicated that gender (HR = 1.111, *P* = 0.437), age (HR = 0.998, *P* = 0.611), stage (HR = 0.985, *P* = 0.804), and risk score (HR = 1.598, *P* < 0.001) were linked to the clinical outcomes of DLBCL patients (Figure 5A), highlighting the significant association between the risk score and the patients’ clinical outcomes. The analysis of multivariate Cox regression indicated that the risk score served as a separate prognostic indicator for DLBCL (Figure 5B). Time-dependent ROC curve showed that AUC values for the 1-, 3- and 5-year survival rates were 0.695, 0.597 and 0.586, respectively (Figure 5C). In addition, the ROC curve showed that the AUC of risk score was 0.694, indicating a clear advantage of the prognostic model over clinical characteristics in terms of predictive capability (Figure 5D).

### Investigation of correlation between risk score and immune infiltration

**Figure 6:**
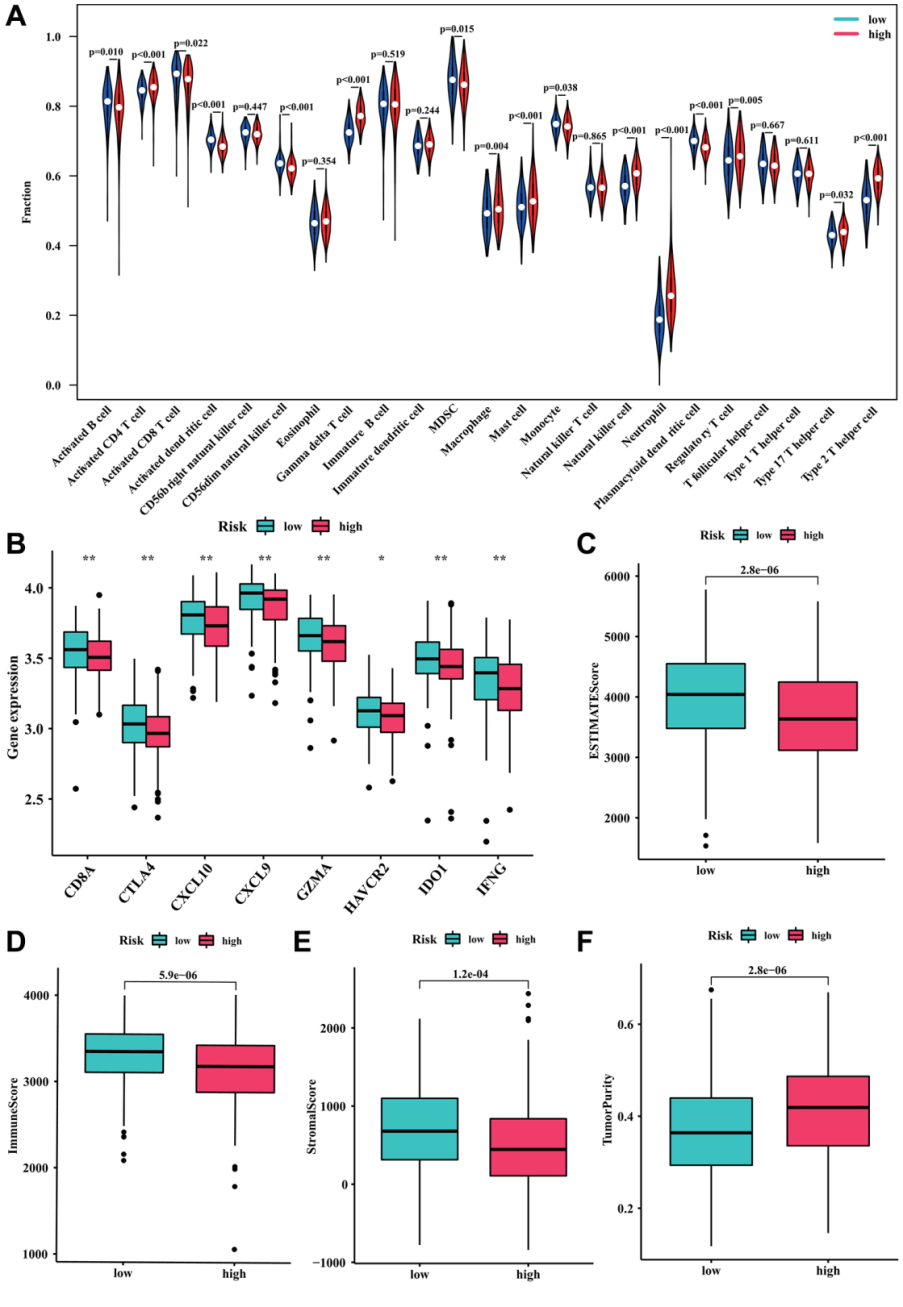
**Relationship between the risk model and immune cell infiltration.** (**A**) Fraction of 23 immune cells in high- and low-risk groups. (**B**) The expression level of immune checkpoints in high- and low-risk groups. (**C**) ESTIMATE score. (**D**) Immune score. (**E**) Stromal score. (**F**) Tumor purity.

The immune infiltration and tumor microenvironment landscape analysis by risk stratification were further explored. The ssGSEA result indicated that the levels of activated CD4+T cell, gamma delta T cell, macrophage, mast cell, natural killer cell, neutrophil, regulatory T cell, type 17 T helper cell, and type 2 T helper cell exhibited a positive correlation with the risk score. Conversely, activated B cell, activated CD8+T cell, activated dendritic cell, CD56dim natural killer cell, MDSC, monocyte, and plasmacytoid dendritic cell were higher in the low-risk group (all *P* < 0.05, Figure 6A). The investigation also examined the variation in immune checkpoints between the two groups at risk. Figure 6B demonstrated that DLBCL patients with a high-risk score showed decreased levels of CD8A, CTLA4, CXCL10, CXCL9, GZMA, HAVCR2, IDO1, and IFNG expression. Furthermore, the findings from the ESTIMATE analysis revealed that DLBCL patients with a low-risk score exhibited elevated ESTIMATE, immune, and stromal scores, while their tumor purity was comparatively lower (Figure 6C–6F).

### Drug sensitivity analysis

**Figure 7:**
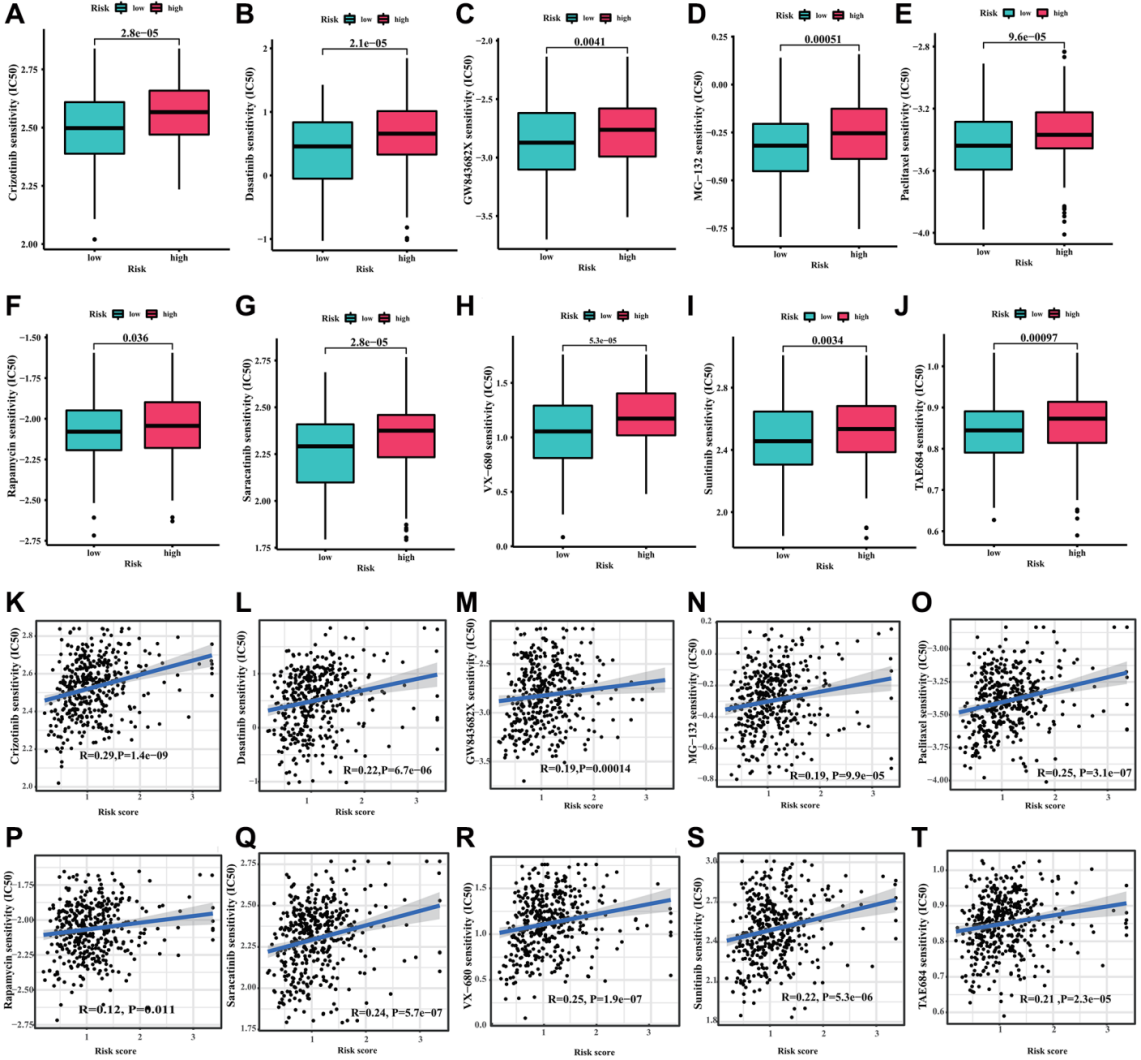
**Drug sensitivity analysis.** (**A**) Crizotinib, (**B**) Dasatinib, (**C**) GW843682X, (**D**) MG-132, (**E**) Paclitaxel, (**F**) Rapamycin, (**G**) Saracatinib, (**H**) VX-680, (**I**) Sunitinib and (**J**) TAE684. (**K**–**T**) Correlation analysis of the risk score and drug sensitivity (IC50).

In order to investigate the potential application of CRIRLs prognostic signature in personalized therapy for DLBCL, we examined the association between the risk score and drug responsiveness. As shown in Figure 7A–7J, the results of drug sensitivity analysis indicated that the IC50 of Crizotinib, Dasatinib, GW843682X, MG-132, Paclitaxel, Rapamycin, Saracatinib, VX-680, Sunitinib and TAE684 were significantly higher in the high-risk group. The risk score was positively related to Crizotinib (R = 0.29, *P* = 1.4e−09), Dasatinib (R = 0.22, *P* = 6.7e−06), GW843682X (R = 0.19, *P* = 0.00014), MG-132 (R = 0.19, *P* = 9.9e−05), Paclitaxel (R = 0.25, *P* = 3.1e−07), Rapamycin (R = 0.12, *P* = 0.011), Saracatinib (R = 0.24, *P* = 5.7e−07), VX-680 (R = 0.25, *P* = 1.9e−07), Sunitinib (R = 0.22, *P* = 5.3e−06) and TAE684 (R = 0.21, *P* = 2.3e−05) (Figure 7K–7T).The findings indicated that these antineoplastic medications may play a promising role in the future treatment of DLBCL.

### qRT-PCR analysis of 4 CRIRLs

**Figure 8:**
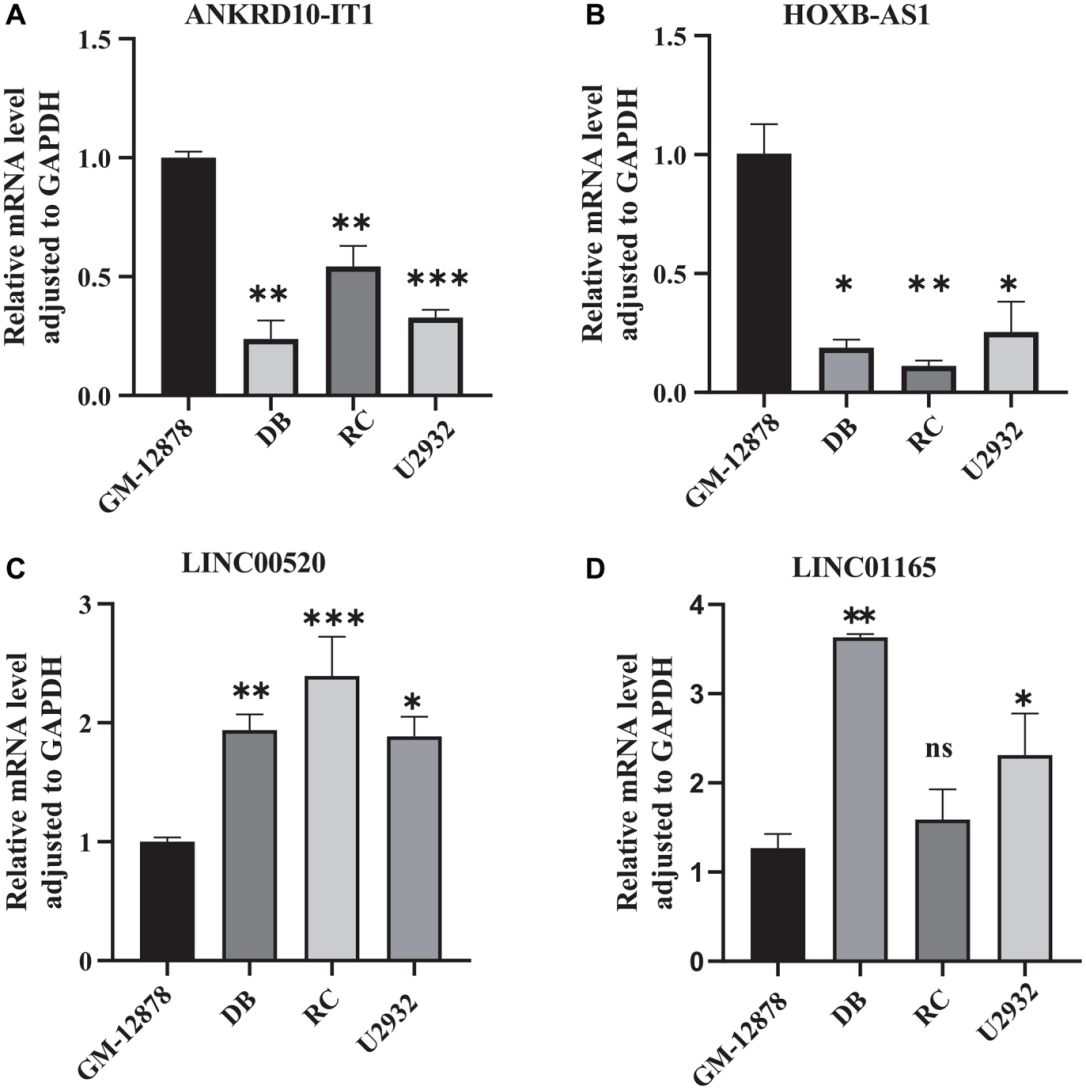
**Validation the expression of CRIRLs.** (**A**) ANKRD10-IT1, (**B**) HOXB-AS1, (**C**) LINC00520, (**D**) LINC01165 expression in human B lymphoid cell line and human DLBCL cell lines. ^*^P < 0.05; ^**^P < 0.01; ^***^P < 0.001.

To further confirm abnormal expressions of the four screened CRIRLs, qRT-PCR was utilized to examine the expressions of 4 CRIRLs in human DLBCL cell lines. As shown in Figure 8, the results exhibited that the mRNA expressions of ANKRD10-IT1 and HOXB-AS1 were obviously down-regulated, while LINC00520 was up-regulated in the human DLBCL cell lines DB, RC and U2932 in comparison to human B lymphoid cell line GM-12878 (Figure 8A–8C). In two of the three DLBCL cell lines, the expression level of LINC01165 was significantly elevated (Figure 8D). These qRT-PCR results verified the reliability of the observations from the public database.

## DISCUSSION

Although significant development in the diagnosis and treatment of DLBCL has been achieved in recent years, the overall DLBCL prognosis still remains unsatisfactory due to complex underlying genetic and molecular mechanisms. Cuproptosis and ICD are two important forms of programmed cell death that play a role in various physiological and pathological processes, including cancer [12, 20]. ICD is a new mode of cell death defined by the NCCA in 2018 that stimulates the tumor-specific immune response to fight against tumor recurrence and metastasis [21]. Cu-based complexes can act as ICD inducers to lead an immune response against tumor cells, which has already demonstrated huge promises as potential new immunotherapies for malignant tumors [17, 22, 23]. Cuproptosis is an innovative approach to cell death that relies on copper. Accumulating evidence suggests that imbalanced copper levels can impact the growth of tumors and lead to various types of cell demise [24], and copper is strongly associated with antitumor immunity and immunotherapy [25]. LncRNAs, which have a crucial impact on the development of cancer, are major contributors to the initiation, advancement, and spread of tumors [26, 27]. Several research studies have indicated that abnormal lncRNA expression is strongly linked to clinical results and could serve as potential prognostic biomarkers in DLBCL [28, 29]. In this study, we discovered four predictive CRIRLs and developed a prognostic risk signature for individuals diagnosed with DLBCL. The utilization of this signature could potentially provide an additional valuable resource to complement the conventional prognostic markers and assist in prognostic forecasting and treatment choices. Furthermore, we explored the immune infiltration landscape and conducted drug sensitivity analysis to understand the underlying mechanism.

The prognostic signature we constructed contains four lncRNAs, ANKRD10-IT1, HOXB-AS1, LINC00520 and LINC01165.One of these tumor types is HOXB-AS1, a cancer stimulator that has been linked to prognosis in various types of tumors [30]. A recent study has shown that HOXB-AS1 plays a part in pathways related to cancer and the immune system, indicating its potential involvement in the development of cancer and immune regulation in various types of cancer [31]. Furthermore, a notable association is observed between HOXB-AS1 and the immune scores in DLBCL, indicating a positive correlation. The expression of HOXB-AS1 also positive associates with the immune checkpoint genes expression level [31]. Our data showed similar results in DLBCL, suggesting that HOXB-AS1 might mediate the role of immune microenvironment in DLBCL.

LINC00520, located at human chromosome 14q22.3, is widely expressed in various tissues and positively correlated with the risk of multiple cancer types. Multiple studies suggest a strong correlation between LINC00520 and the clinicopathological features, prognosis, and therapy response in cancer patients, indicating the significant potential of LINC00520 in cancer diagnosis, prognostic prediction, and treatment [32, 33]. LINC00520 could function as an oncogene that generates oncogenic transformation. Furthermore, LINC00520 has a crucial function in controlling the PI3K/AKT and JAK/STAT signaling pathways [33]. Intriguingly, it has been reported that PI3K/AKT signaling pathway is identified as a crucial regulator of proliferation and survival of tumor cells in DLBCL, and therefore targeting the signaling might be a promising therapeutic intervention for DLBCL patients [34, 35]. The JAK/STAT signaling pathways play a role in numerous biological processes, including the proliferation, migration, invasion, and angiogenesis of tumor cells. In a whole exome sequencing and high-throughput second-generation sequencing study of DLBCL patients, a large number of carcinogenic mutations are observed in JAK/STAT signaling pathway, resulting in hyperactive pathway [36]. Similar results are obtained in another study. In EBV-positive HIV-associated DLBCL, mutations in JAK/STAT pathway also leads to abnormal activation and elevated expression of downstream oncogenes, such as c-MYC, thereby resulting in the progression of malignant tumors [37, 38]. In addition, the dissemination of DLBCL could be facilitated by STAT3-coordinated migration [39]. The results of these studies suggests that activation of the signaling pathways may lead to the occurrence and development of DLBCL [40]. In summary, we hypothesize that LINC00520 may be contribute to tumorigenesis and cancer development of DLBCL by PI3K/AKT and JAK/STAT signaling pathways. Additionally, LINC00520 could serve as a possible indicator and a hopeful treatment target for DLBCL.ANKRD10-IT1 has been utilized as a diagnostic or prognostic indicator in various cancer types [41, 42]. Currently, there is limited knowledge regarding the role of LINC01165 in biological processes. Hence, additional empirical research will be necessary in the coming times.

Immune regulation plays an essential role in the progression of DLBCL, and immunotherapy is an important treatment for DLBCL [43]. Hence, it is crucial to investigate immune infiltration in order to elucidate the connection between tumors and the immune system. The current investigation revealed that patients with high-risk DLBCL had increased infiltration of regulatory T cells (Tregs) and neutrophils. The suppression of effector CD8+ T cell proliferation and cytokine production by Treg can hinder the effectiveness of antitumor immune responses, potentially leading to an inadequate antitumor response and promoting the growth of cancer cells [44, 45]. Furthermore, the predictive impact of Treg remains a subject of debate in DLBCL, with certain investigations indicating that the quantity and ratio of infiltrating Treg are linked to an unfavorable prognosis [44, 45], aligning with our findings. Tumor-infiltrating neutrophils are strongly associated with tumor growth and metastasis [46]. The aforementioned findings indicate that the inferior outlook of high-risk individuals can be attributed to increased immunosuppression within the tumor microenvironment, potentially playing a role in the advancement of the tumor. Furthermore, our investigation revealed that individuals in the low-risk category exhibited reduced resistance to antineoplastic medications, including conventional chemotherapy drugs, Dasatinib, Paclitaxel and Rapamycin [47–49]. Some new drugs also exhibited great potential as anticancer therapy to DLBCL, such as MG132 and VX-680 [50, 51], suggesting the impact of the novel risk signature on risk stratification and medication selection reference.

To summarize, we have effectively created a prognostic marker for DLBCL by utilizing four lncRNAs associated with cuproptosis and immunogenic cell death. This discovery holds potential as both a biomarker and a therapeutic target, influencing the advancement of DLBCL. Additionally, an examination was conducted on the landscape of immune infiltration and the analysis of drug sensitivity. These investigations can potentially enhance our comprehension of the relationship between cuproptosis and immunogenic cell death. Moreover, this research could offer fresh insights and guidance for accurate therapeutic measures in DLBCL.

## Supplementary Materials

Supplementary Tables 1 and 2

Supplementary Table 3

Supplementary Table 4

Supplementary Table 5

Supplementary Table 6
