# Supplementary material for: Development and validation of a combined cuproptosis and immunogenic cell death prognostic model for diffuse large B-cell lymphoma
Source: Aging (Albany NY). 2024 Jan 26;16(2):1218–36. doi: 10.18632/aging.205399 (PMC10866411; doi:10.18632/aging.205399)
Supplement: Supplementary Tables 1 and 2 [file aging-16-205399-s001.pdf]

SUPPLEMENTARY TABLES

Supplementary Table 1. The 10 cuproptosis-related genes.

FDX1
LIAS
LIPT1
DLD
DLAT
PDHA1
PDHB
MTF1
GLS
CDKN2A

Supplementary Table 2. The 33 immunogenic cell death-related genes.

IL17RA
IL1R1
PIK3CA
CD4
IFNG
PRF1
CXCR3
CD8A
CD8B
P2RX7
NLRP3
IL10
TLR4
ENTPD1
ATG5
IFNB1
IL6
EIF2AK3
IL17A
LY96
FOXP3
HMGB1
HSP90AA1
BAX
PDIA3
CALR
CASP8
MYD88
IFNGR1
CASP1

IL1B
TNF
NT5E
